# The Effect of Jazar Supplement on Quality of Life and Sexual Function in Postmenopausal Women: A Double-Blind, Randomized, Placebo-Controlled Trial

**DOI:** 10.1155/2021/8854182

**Published:** 2021-02-25

**Authors:** Sousan Hafizi, Alireza Abbassian, Malihe Tabarrai

**Affiliations:** Department of Traditional Medicine, School of Persian Medicine, Tehran University of Medical Sciences, Tehran, Iran

## Abstract

**Background:**

Menopause is one of the most critical stages of a woman's life and is accompanied by symptoms including hot flashes, night sweats, mood swings, sexual dysfunction, mucus atrophy, and vaginal dryness. Women tend to use complementary and alternative medicine such as herbs rather than hormone replacement therapy to alleviate these symptoms. The purpose of this study is to examine the effects of Jazar supplement (herbal supplement comprising Vitex, fennel, and carrot seeds) on sexual function, quality of life, and vaginal atrophy in postmenopausal women.

**Methods:**

This study was a randomized clinical trial conducted on ninety postmenopausal women. Participants were randomized to intervention/control groups using block randomization. The intervention group received four Jazar capsules (500 mg each) daily, while the control group received a placebo for eight weeks. Data were gathered using a socio-demographic questionnaire, the Female Sexual Function Index (FSFI), and the Menopause-Specific Quality of Life (MENQOL) before the intervention and at weeks 4, 8, and 10. Vaginal pH and vaginal maturation index (VMI) were measured before and at the end of the study.

**Results:**

Eighty-four women completed the trial, and six women withdrew. There were no remarkable differences between the two groups in terms of quality of life, sexual function, VMI, and vaginal pH at baseline. At the end of the study, participants in the intervention group had a significantly lower score in terms of quality of life (*P* < 0.001) and vaginal pH (*P*=0.001), and a higher FSFI (*P* < 0.001) and VMI (*P*=0.030) score compared to the control group.

**Conclusion:**

Based on the findings of the present study on menopausal women, the Jazar supplement significantly improved quality of life and sexual function and prevented or delayed vaginal atrophy.

## 1. Introduction

Menopause is one of the most critical stages of a woman's life determined by changes in the level of sexual hormones, cessation of menstruation, and inability to conceive [1]. This phenomenon usually occurs between 45 and 60 years of age [2]. The mean age of menopause is reported 48.2 in Iran [3], while estimated to be approximately 51 in developed countries [4]. The number of postmenopausal women is estimated to rise from 742 million in 2010 to 1.2 billion by 2030, with the highest increase in developing countries [5].

Menopause is a normal physiological condition that induces both physical and psychological changes, thereby influencing the quality of life [6]. Some common postmenopausal symptoms include hot flashes, sleep disorders, night sweats, mood swings, cognitive problems, sexual dysfunction, mucus atrophy, dry vagina, and weight gain [7]. These symptoms are experienced by over 85% of women in the menopausal stage [8] and usually last for 5 to 7 years but can continue for 15 years or more [9].

Hormonal replacement therapy (HRT) is typically considered a primary treatment to improve the symptoms of menopause [10]. However, it is accompanied by side effects, such as the increased risk of cardiovascular diseases, breast and endometrial cancers, thromboembolism, vaginal bleeding, and liver diseases [11]. On account of the mentioned concerns on the one hand, and the lower side-effect, higher accessibility, and lower costs of herbal medicine, particularly in developing countries, on the other hand, there is a growing trend to use complementary and alternative medicine (CAM) [11, 12]. About 51% of women use CAM in menopause, with more than 60% confirming the efficacy of CAM in the alleviation of symptoms [13]. These women use different CAM methods, including relaxation, meditation, aromatherapy, traditional Chinese medicine (TCM), reflexology, acupuncture, vitamins, minerals, dietary supplements, and herbs [14].

Among the various methods of CAM, herbal medicine is the most popular and fast-growing modality [15]. Evidence from numerous studies supports the effectiveness of a broad spectrum of herbs in some postmenopausal symptoms [16]. Moreover, traditional Persian medicine (TPM) introduces various herbs that could be useful in treating menopausal symptoms, with evidence in favor of their efficacy based on the findings of several prior investigations [17]. Jazar is a TPM formula comprising Vitex (*Vitex agnus-castus*), fennel (*Foeniculum vulgare* Mill), and carrot (*Daucus carota* subsp*. sativus*) seeds. This formula was recommended for treatment of menopause symptoms and low libido based on TPM references such as “Canon of Medicine” written by Avicenna and the book of “Qarabadineh Kabir” written by Aghil Shirazi.

Although some investigations have evaluated Vitex or fennel's effect on either postmenopausal symptoms or sexual dysfunction alone, no study has been dedicated to assessing the effects of Jazar (coadministration of Vitex, fennel, and carrot seeds) on menopausal symptoms and sexual dysfunction. Therefore, the purpose of the present study is to explore the efficacy and safety of Jazar on menopausal symptoms and sexual dysfunction among postmenopausal women.

## 2. Methods

### 2.1. Trial Design

A 10-week, multi-center, randomized, double-blind, placebo-control trial was performed in the outpatient clinics of Farmanfarmaian and Abouzar affiliated to Tehran University of Medical Sciences between January 2019 and October 2019.

The protocol of the study was confirmed by the Review Board and the Ethical Committee of Tehran University of Medical Sciences (number: IR.TUMS.VCR.REC.1395.1531) and followed the Declaration of Helsinki and its subsequent revisions. The trial was registered at the Iranian Registry of Clinical Trials on October 9, 2018 (https://www.irct.ir/trial/32481; registration number: IRCT20180705040349N1). All eligible participants signed a written consent form before the initiation of intervention. The purpose, procedure, benefits, and risks of the trial were explained to the participants, and they were informed about their legal right to withdraw from the trial at any stage of intervention. We conformed to the CONSORT  checklist as the guideline for reporting this study (see Appendix S1).

### 2.2. Participants

We recruited postmenopausal women aged between 45 and 60 with a Female Sexual Function Index (FSFI) score of ≤26. Participants who met the following criteria were included: no menstruation in the last 12 months and duration of menopause <5 years, being married and having a steady sexual partner, not taking hormone therapy over the previous six months, not using alcohol, nonsmoking, undergoing natural menopause, hot flash attacks of ≥2 times per day, and normal Pap smear test during the last two years. Patients were excluded if they had chronic diseases such as cardiovascular diseases, diabetes, and cancer; psychological disorders such as severe depression and anxiety; or allergy to herbal medicine. They were also excluded from the study if they had used any medications or supplements to treat symptoms related to menopause during the last four weeks.

### 2.3. Sample Size Estimation

Based on prior similar studies [18] and considering *p*_1_ = 20%, *p*_0_ = 50%, *α* = 0.05, *β* = 20%, and sample size equation for comparing two populations, the sample size was calculated for about 40 individuals. Finally, assuming a 10% attrition rate, 45 patients were recruited in each group:

(1) $$\begin{array}{c} N=\frac{2{\left(Z_{1-\alpha /2}+Z_{1-\beta }\right)}^{2}\times p\left(1-p\right)}{{\left(p_{0}-p_{1}\right)}^{2}},\\ P=\frac{p_{0}+p_{1}}{2}. \end{array}$$

### 2.4. Randomization and Intervention

Using the permuted block technique, participants were randomly assigned to Jazar and placebo groups with blocks of size four and a 1 : 1 allocation ratio. The allocation sequence was generated by a computer using a random numbers table. An independent person conducted randomization, and participants and investigators were concealed. Patients in the intervention group received four 500 mg capsules of Jazar twice per day (two capsules after breakfast and two capsules after dinner), whereas the patients in the control group received matched placebo capsules for 8 weeks. Jazar capsules contained 500 mg of fennel, Vitex, and carrot seeds equally, while placebo capsules contained 500 mg of starch. Jazar capsules were prepared by Green Gold of the Tooba company and approved by the Iranian Food and Drug Administration (registry code: 462264). There was no detectable difference between Jazar and placebo capsules in terms of size, shape, color, odor, and texture.

Phone calls to participants were made to remind regular intake of capsules and the next visit's date. Moreover, a TPM practitioner responded to any questions regarding intervention throughout the study. Furthermore, adherence to drug intake was monitored by weekly phone calls. Patients were followed for two weeks after completion of the eight-week intervention.

### 2.5. Outcomes

The instruments used for data collection consisted of a socio-demographic questionnaire, the Female Sexual Function Index (FSFI), the Menopause-Specific Quality of Life (MENQOL) questionnaire, vaginal pH measurement, vaginal maturation index, and a checklist of side effects. The socio-demographic questionnaire including age, height, weight, age at menarche, marriage, pregnancy and menopause, marital status, method of delivery, number of offsprings, income, education, occupation, age of spouse, education, and occupation was obtained from participants before the initiation of intervention.

The Self-Reported Female Sexual Function Index (FSFI) questionnaire [19, 20] was used to evaluate the sexual function at baseline and at weeks 4, 8, and 10. A valid and reliable 19-item self-report Likert-type questionnaire, FSFI, covers six dimensions of women's sexual function, including sexual desire (2 items), sexual arousal (4 items), lubrication (4 items), orgasm (3 items), satisfaction (3 items), and pain related to vaginal penetration (3 items). The total score lies in a range of 2 to 36, with higher scores representing a more favorable sexual function.

The Menopause-Specific Quality of Life (MENQOL) [21, 22] is a valid, reliable, and self-administered questionnaire designed to assess the quality of life in menopausal women. Participants completed this questionnaire at baseline and also at weeks 4, 8, and 10. This instrument contains 29 questions with a 7-point Likert scale (0 = not bothered at all to 6 = too bothered) and assesses four aspects of vasomotor (3 items), psychosocial (7 items), physical (16 items), and sexual functioning (3 items). Higher scores indicate lower quality of life in menopausal women.

Furthermore, the Pap smear test was performed to determine the vaginal maturation index (VMI). VMI was calculated based on the following formula: VMI = 0.5 × (percent intermediate cells) + percent superficial cells. Moreover, vaginal pH was measured by pH-indicator strips (MColorpHast™ Nonbleeding pH-Indicator Strips). VMI and vaginal pH are appropriate tools for diagnosing vaginal atrophy [23, 24]. The Pap smear test and vaginal pH measurement were conducted at the beginning and end of the study (week 10).

A checklist was used by a practitioner during each follow-up visit to evaluate the safety of medications and potential side effects experienced by participants. It consisted of items on vaginal bleeding, diarrhea or digestive symptoms, hypertension, breast enlargement, mood problems, etc.

### 2.6. Statistical Analyses

SPSS version 19 (SPSS Inc., Chicago, IL, USA) was used for statistical analysis. *P* values 0.05 or below were regarded as significant. In assessing demographic details, categorical variables were reported as percentages (%), whereas mean ± SD was used for continuous data. To examine homogeneity regarding socio-demographic characteristics, the chi-square test was used for categorical variables and one-way analysis of variance (ANOVA) for continuous variables. An independent *t*-test was applied to compare FSFI, MENQOL, vaginal pH, and VMI between intervention and control groups.

## 3. Results

**Figure 1:**
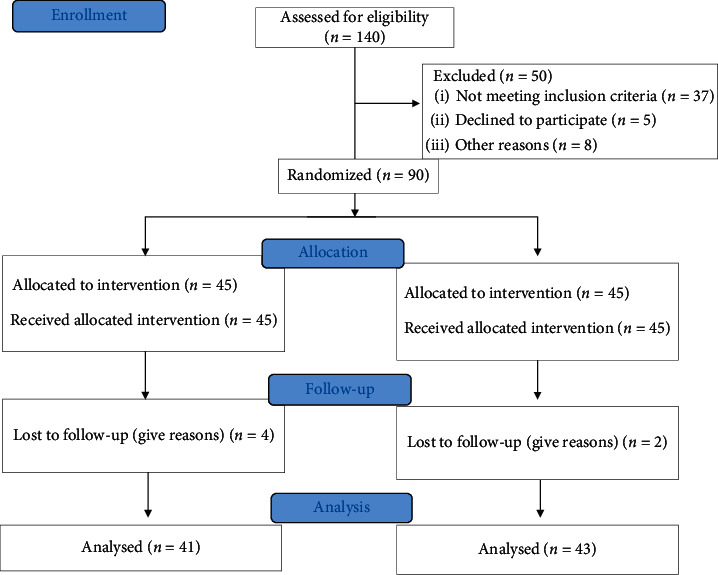
Flow diagram of the study.

Among the 140 women examined for eligibility, ninety participants met the inclusion criteria and were randomized to receive either Jazar (*n* = 45) or placebo (*n* = 45). At the end of the study, six women (2 in the intervention group and 4 in the placebo group) dropped out, and 84 participants completed the study (Figure 1). The general characteristics of participants in intervention and control groups are shown in Table 1. The mean age of participants was 52.40 and 54.49 years in intervention and control groups, respectively, which was indicative of a significant difference between the two groups (*P*=0.020). Moreover, the mean age of the spouse in the control group was higher than the intervention group (*P*=0.015). No significant difference was observed between the two groups in any of the other baseline characteristics.

Table 2 reveals the mean score of MENQOL subscales (vasomotor, psychosocial, physical, and sexual) and the overall MENQOL score of two groups at weeks 0, 4, 8, and 10. No significant difference existed between the two groups in the overall MENQOL score nor any subscales at baseline. In comparison, the mean score of overall MENQOL score and all subscales was significantly lower than the placebo group at weeks 4, 8, and 10 (*P* < 0.05).

The total FSFI score was 14.38 ± 9.41 in the intervention group and 10.89 ± 8.49 in the control group at the beginning of the study, indicating no significant difference (*P*=0.124). Moreover, there was no significant difference in any FSFI domain (desire, arousal, lubrication, orgasm, satisfaction, and pain) between the intervention and placebo groups. However, at the end of weeks 4, 8, and 10, women in the intervention group had a significantly higher score in comparison with the placebo group (*P* < 0.05) (Table 3).

The changes in VMI and vaginal pH are shown in Table 4. No significant difference was observed between the two groups before intervention (6.19 ± 1.07 vs. 6.24 ± 1.06). However, participants in the intervention group had lower vaginal pH compared to women in the control group at the end of treatment. Furthermore, results indicated that there was no significant difference in baseline VMI between the two groups, but the difference among the groups became significant at the end of week 10 (*P*=0.03). Throughout the study, no significant side effects were reported from either group.

## 4. Discussion

This research was designed to investigate the Jazar supplementation effect on postmenopausal symptoms, sexual dysfunction, and vaginal atrophy in menopausal women. Our findings demonstrated that compared to the placebo, Jazar supplementation decreased postmenopausal symptoms and improved all areas of sexual function and vaginal atrophy in participants.

Menopause is due to estrogen deficiency and is accompanied by bothersome symptoms such as night sweats, hot flushes, insomnia, sexual dysfunction, and vaginal atrophy [25]. Phytoestrogens are botanical ingredients with estrogen-like features [26]. Some meta-analyses of the effects of phytoestrogens on menopausal symptoms and sexual function have shown a significant reduction in the frequency of hot flashes and an improvement in sexual disturbances. However, phytoestrogen consumption has not been demonstrated to have a considerable effect on the Kupperman Index (KI), which consists of eleven menopausal symptoms, including vasomotor signs, melancholia, paresthesia, sleep disturbances, mood disorders, vertigo, arthralgia/myalgia, weakness, headache, formication, and palpitations [27, 28]. Differences in participant numbers, outcomes and phytoestrogen dosages, heterogeneity among the eligible studies, and the low number of pooled analyses may be the reason for the null findings [27].

Fennel, Vitex, and carrot seeds are good sources of phytoestrogens. The medicinal plant used worldwide, fennel (*Foeniculum vulgare* Mill), contains flavonoids (flavonoid glycosides and flavonoid aglycons), phenolic compounds, phenolic acids, hydroxycinnamic acids, coumarins, and tannins with antioxidant properties [29]. Phytoestrogenic compounds in fennel play a role in improving premenstrual symptoms, menopausal symptoms, sexual activity, vaginal atrophy, amenorrhea, dysmenorrhea, lactation, and polycystic ovary syndrome [30, 31].

Vitex (*Vitex agnus-castus*), mostly found in the Mediterranean region, has many applications in traditional medicine. Flavonoids (vitexin, casticin), iridoid glycoside (agnuside, aucubin), alkaloids, diterpenoids, p-hydroxybenzoic acid, and steroids are chemical compounds of Vitex [32]. Studies have reported therapeutic uses of Vitex on menopausal symptoms, infertility, premenstrual syndrome, and hyperprolactinemia. Possible mechanisms of action in reducing menopausal symptoms include stimulation of the dopaminergic system, binding to opioid receptors, and increasing melatonin secretion [33].

Carrot (*Daucus carota* subsp*. sativus*) seeds have been mentioned in various European publications as a contraceptive, aphrodisiac, emmenagogue, and antifertility agent [34]. Estrogenic or antiprogestogenic activities may be a potential mechanism for this plant's abortive effect [35]. A study conducted by Bhatnagar et al. showed antiestrogenic activity for carrot seed extract at lower dosages (3–100 mg/kg BW), while higher doses (150–250 mg/kg BW) lead to an estrogenic response in rats [36].

In a randomized, placebo-controlled clinical trial published in 2017, the effect of an 8-week supplementation with fennel (100 mg/d) on menopausal symptoms was investigated in 90 postmenopausal Iranian women. This study demonstrated that fennel supplementation could decrease the Menopause Rating Scale (MRS) significantly at 4, 8, and 10 weeks (2 weeks after intervention) compared to the placebo [37].

The low blood flow in the vulvovaginal region, caused by a decrease in estrogen and androgen concentrations, plays a pivotal role in reducing sexual arousal and libido in menopausal women. In one RCT  study, 60 postmenopausal women received fennel (5 g/day) and placebo as vaginal cream for eight weeks. All aspects of sexual activity, including lubrication, arousal, sexual satisfaction, orgasm, and pain, except for burning, improved significantly in the fennel group compared to the placebo group [38]. Furthermore, despite the insignificant change in burning, all vaginal atrophy symptoms such as dryness, itching, and pallor improved significantly in the fennel group compared to the placebo group [39].

In a study conducted by Rahimikian et al. [37], 52 women were randomly assigned to use 90 mg of fennel oil or sunflower oil per day for eight weeks. Fennel consumption had no significant effects on vaginal atrophy criteria, such as the number of superficial, intermediate and para-basal cells, vaginal pH, and vaginal maturation index. According to the results of two clinical studies, fennel vaginal cream improves vaginal atrophy in postmenopausal women, while oral fennel oil has no effects on this symptom. More clinical trials are necessary to verify the efficacy of different modes of fennel administration on vaginal atrophy.

The results of one RCT  conducted in Iran indicated that the daily use of 2 grams of fennel seed powder for 8 weeks significantly improves menopausal symptoms in postmenopausal women, while its effect on estradiol levels and sexual activity is not significant. However, another RCT in Iran reported that 100 mg capsules containing 30% fennel for three months in fifty postmenopausal women do not lead to significant improvement in menopausal symptoms, except for stress incontinence [40]. A high placebo response may be the reason for nonsignificant results.

In a double-blind clinical trial in 2011, Abbaspoor et al. [41] demonstrated that vasomotor symptoms during menopause are relieved by consuming Vitex supplementation. In another RCT on menopausal women, an 8-week supplementation with 30 mg Vitex decreased the mean scores for the total menopausal disorder, anxiety, and vasomotor dysfunction compared to the placebo. However, other menopausal symptoms such as somatic complications, depression, and sexual dysfunction were not significantly different between the Vitex and placebo groups [42].

Although some researches have focused on Vitex or fennel's effect on postmenopausal symptoms, sexual dysfunction, and vaginal atrophy, the synergistic effect that may exist between these herbs in the form of a formula needed investigation. This is the first randomized clinical trial that assessed the effects of Jazar (combination of Vitex, fennel, and carrot seeds) supplementation on postmenopausal symptoms, sexual dysfunction, and vaginal atrophy. The findings of this study may be implicated for women with menopausal symptoms who decline or are not suitable cases for hormone replacement therapy. The limitations of this study include (1) small sample size; (2) lack of measurement of serum estrogen levels; and (3) respondent bias that resulted from using a questionnaire to evaluate menopausal symptoms and sexual function. However, the results of vaginal atrophy are relatively accurate because they have been assessed using laboratory tests.

In conclusion, this study demonstrated that Jazar supplements decrease postmenopausal symptoms and improve sexual dysfunction and vaginal atrophy in menopausal women. Further randomized clinical trials with a higher sample size and longer treatment duration are needed to confirm our findings.

## Figures and Tables

**Table 1:** Baseline characteristics of participants.

Variables	Jazar (*n* = 43)	Placebo (*n* = 41)	*P* value
Age (years)	52.40 ± 4.20	54.49 ± 3.83	0.020^a^
BMI (kg/m^2^)	27.67 ± 3.78	27.66 ± 3.51	0.995
Menarche (years)	12.79 ± 1.34	13.27 ± 1.41	0.114
Menopause (years)	46.28 ± 6.92	48.49 ± 4.54	0.087
Age of marriage (years)	19.72 ± 4.79	19.02 ± 3.90	0.466
Age of first labor (years)	21.84 ± 5.14	21.27 ± 4.20	0.579
Spouse age (years)	56.44 ± 6.49	59.78 ± 5.80	0.015
Education			
Under diploma	2 (66.7%)	1 (33.3%)	0.398^b^
Diploma	21 (58.3%)	15 (41.7%)	
Above diploma	20 (44.6%)	25 (55.6%)	
Job status			
Negative	35 (47.9%)	38 (52.1%)	0.125
Positive	8 (72.7%)	3 (27.3%)	
Children number			
0–3	33 (47.1%)	37 (52.9%)	0.097
4–8	10 (71.4%)	4 (28.6%)	
Spouse education			
Under diploma	1 (50%)	1 (50%)	0.182
Diploma	21 (63.6%)	12 (36.4%)	
Above diploma	21 (42.9%)	28 (57.1%)	
Spouse job status			
Negative	24 (54.5%)	20 (45.5%)	0.519
Positive	19 (47.5%)	21 (52.5%)	

Categorical variables are represented as percentage (%) and continuous data are represented as mean ± SD. ^a^One-way analysis of variance (ANOVA). ^b^Chi-square for trend test.

**Table 2:** A summary of changes in the scores of the MENQOL subscales during the trial period.

Subscales	Measurement points	Jazar (*n* = 43)	Placebo (*n* = 41)	*P* value
Vasomotor	Baseline	3.46 ± 1.59	3.21 ± 1.34	0.433
	Week 4	2.24 ± 1.27	3.24 ± 1.29	0.001
	Week 8	1.61 ± 1.20	2.76 ± 1.14	<0.001
	Week 10	1.59 ± 1.20	2.62 ± 1.42	0.001

Psychosocial	Baseline	2.58 ± 1.49	2.18 ± 1.17	0.177
	Week 4	2.23 ± 1.29	3.18 ± 1.21	0.001
	Week 8	2.04 ± 1.24	2.49 ± 1.07	0.079
	Week 10	2.03 ± 1.25	1.24 ± 2.69	0.017

Physical	Baseline	2.79 ± 1.18	2.42 ± 1.29	0.178
	Week 4	2.47 ± 1.12	3.42 ± 1.07	<0.001
	Week 8	2.29 ± 1.08	2.69 ± 1.17	0.109
	Week 10	2.28 ± 1.09	2.92 ± 1.09	0.009

Sexual	Baseline	4.03 ± 1.57	3.66 ± 1.77	0.311
	Week 4	3.37 ± 1.56	4.30 ± 1.41	0.005
	Week 8	3.04 ± 1.39	3.71 ± 1.46	0.033
	Week 10	3.01 ± 1.38	3.75 ± 1.44	0.020

Total	Baseline	3.22 ± 0.99	2.87 ± 0.98	0.108
	Week 4	2.58 ± 0.87	3.53 ± 0.79	<0.001
	Week 8	2.24 ± 0.88	2.91 ± 0.78	<0.001
	Week 10	2.23 ± 0.88	2.99 ± 0.88	<0.001

The data are presented as mean (standard deviation). For comparison of the groups, an independent *t*-test was used.

**Table 3:** A summary of changes in the scores of the FSFI subscales during the trial period.

Subscales	Measurement points	Jazar (*n* = 43)	Placebo (*n* = 41)	*P* value
Desire	Baseline	2.10 ± 1.31	1.56 ± 1.32	0.063
	Week 4	2.83 ± 0.85	2.04 ± 1.24	0.001
	Week 8	3.23 ± 0.74	2.22 ± 1.48	<0.001
	Week 10	3.12 ± 0.84	1.97 ± 1.56	<0.001

Arousal	Baseline	1.96 ± 1.35	1.40 ± 1.31	0.057
	Week 4	2.77 ± 1.07	1.91 ± 1.39	0.002
	Week 8	3.04 ± 0.95	1.90 ± 1.49	<0.001
	Week 10	2.95 ± 1.05	1.92 ± 1.49	<0.001

Lubrication	Baseline	2.25 ± 1.75	1.83 ± 1.68	0.607
	Week 4	3.43 ± 1.50	2.34 ± 1.63	0.002
	Week 8	3.60 ± 1.53	2.15 ± 1.77	<0.001
	Week 10	3.59 ± 1.55	2.13 ± 1.86	<0.001

Satisfaction	Baseline	2.43 ± 1.89	1.79 ± 1.69	0.270
	Week 4	3.55 ± 1.48	2.44 ± 1.76	0.002
	Week 8	3.86 ± 1.27	2.53 ± 1.81	<0.001
	Week 10	3.67 ± 1.57	2.44 ± 1.93	0.002

Orgasm	Baseline	2.91 ± 1.93	2.23 ± 1.73	0.106
	Week 4	4.05 ± 1.40	2.90 ± 1.80	0.002
	Week 8	4.25 ± 1.28	2.78 ± 1.92	0.003
	Week 10	4.16 ± 1.41	2.89 ± 2.01	0.001

Dyspareunia	Baseline	2.70 ± 2.13	2.05 ± 1.64	0.095
	Week 4	3.97 ± 1.63	2.87 ± 1.85	0.005
	Week 8	4.16 ± 1.67	2.77 ± 1.96	0.001
	Week 10	4.11 ± 1.75	2.55 ± 2.19	0.001

Total	Baseline	14.38 ± 9.41	10.89 ± 8.49	0.124
	Week 4	20.63 ± 6.72	14.54 ± 8.60	0.005
	Week 8	22.17 ± 6.28	14.37 ± 9.82	0.001
	Week 10	21.63 ± 7.02	13.93 ± 10.31	<0.001

The data are presented as mean (standard deviation). For comparison of the groups, an independent *t*-test was used.

**Table 4:** A summary of changes in the vaginal pH and vaginal maturation index (VMI) during the trial period.

Subscales	Measurement points	Jazar (*n* = 43)	Placebo (*n* = 41)	*P* value
Vaginal pH	Baseline	6.19 ± 1.07	6.24 ± 1.06	0.805
	Week 10	5.74 ± 0.82	6.49 ± 1.12	0.001

VMI	Baseline	39.63 ± 24.05	36.76 ± 23.30	0.057
	Week 10	44.46 ± 21.91	33.89 ± 21.82	0.030

The data are presented as mean (standard deviation). For comparison of the groups, an independent *t*-test was used.

## Data Availability

The data used to support the findings of this study are available from the authors.
